# Knockdown of annexin A5 restores gefitinib sensitivity by promoting G2/M cell cycle arrest

**DOI:** 10.1186/s12931-018-0804-1

**Published:** 2018-05-21

**Authors:** Jian Zhou, Meijia Chang, Jing Li, Tao Fang, Jie Hu, Chunxue Bai

**Affiliations:** 1 Department of Pulmonary Medicine, Zhongshan Hospital, Fudan University, Shanghai, China; 2 Department of Oncology, Shengli Oilfield Central Hospital, Shandong Province, China; 3 Shanghai Respiratory Research Institute, Shanghai, China; 4 State Key Laboratory of Respiratory Disease, Guangzhou Medical University, Guangzhou, China

**Keywords:** Non-small cell lung cancer, Annexin A5, Gefitinib, Polo-like kinase 1

## Abstract

**Background:**

Epidermal growth factor receptor (EGFR) tyrosine kinase inhibitors, including gefitinib, are first-line drugs against advanced non-small cell lung cancer with activating EGFR mutations. However, the development of resistance to such drugs is a major clinical challenge.

**Methods:**

The role of annexin A5 in resistance to EGFR tyrosine kinase inhibitors was investigated by qPCR and western blot of relevant molecules, by CCK8 and EdU assay of cell proliferation and viability, by annexin V/propidium iodide assay of apoptosis and cell cycle distribution, by JC-1 assay of mitochondrial integrity, and by xenograft assay of tumorigenicity.

**Results:**

We found that annexin A5 is upregulated in gefitinib-resistant cell lines, as well as in clinical specimens resistant to EGFR tyrosine kinase inhibitors. Accordingly, knockdown of the gene from gefitinib-resistant cells restores gefitinib sensitivity in vitro and in vivo by downregulating polo-like kinase 1 signal pathway, thereby inducing mitochondrial damage, caspase activation, cell cycle arrest at G2/M, and, finally, apoptosis.

**Conclusions:**

The data indicate that annexin A5 confers gefitinib resistance in lung cancer by inhibiting apoptosis and G2/M cell cycle arrest, and is thus a potential therapeutic target in non-small cell lung cancers resistant to EGFR tyrosine kinase inhibitors.

## Background

Lung cancer is highly aggressive and is the leading cause of cancer-related mortality worldwide. Non-small cell lung cancer, which comprises 80–85% of all lung cancer cases, is also one of the most common malignancies overall, with adenocarcinoma as the main histological type [1]. Since the discovery of epidermal growth factor receptor (EGFR) activating mutations in non-small cell lung cancer, and the subsequent success of EGFR tyrosine kinase inhibitors, treatment of advanced cases has shifted from chemotherapy to molecular-targeted therapy, especially in Asians, females, never smokers, and/or patients with adenocarcinoma, of whom about 40–50% harbor a mutation in the tyrosine kinase domain in EGFR [2]. Indeed, EGFR tyrosine kinase inhibitors, which suppress cancer cell proliferation and induce apoptosis, cell cycle arrest, and senescence, are now standard therapy for advanced cases with activating EGFR mutations. Nevertheless, progression-free survival is less than 1 year for patients treated with these inhibitors [3, 4], and mechanisms of resistance remain obscure despite extensive research.

Annexin A5 (ANXA5), which contains a very short unphosphorylated N-terminus in comparison to other annexins, regulates cell proliferation, signal transduction, and coagulation [5–7]. In particular, ANXA5 binds to cell membranes rich in phosphatidylserine or in response to a Ca^2+^ burst, and promotes membrane resealing by forming two-dimensional arrays at the site of membrane damage [8, 9]. Strikingly, ANXA5 is overexpressed in many cancers, including renal cell cancer, hepatocellular carcinoma, colorectal cancer, and breast cancer [10–13]. Intriguingly, Lu et al. found that the natural product berberine may target ANXA5 in ovarian cancer, and, conversely, ANXA5 may block the antitumor activity of berberine [14]. However, the role of ANXA5 in non-small cell lung cancer, and especially in resistance to EGFR tyrosine kinase inhibitors, is poorly understood.

We now report that ANXA5 is upregulated in gefitinib-resistant cell lines, as well as in clinical specimens resistant to EGFR tyrosine kinase inhibitors. In particular, ANXA5 promotes gefitinib resistance by inhibiting apoptosis and cell cycle arrest. Indeed, knockdown of ANXA5 from gefitinib-resistant cells restores sensitivity to the drug. Taken together, the data highlight ANXA5 as a mediator of resistance to EGFR tyrosine kinase inhibitors.

## Methods

### Cell culture

PC9 and H4006 human lung adenocarcinoma cells were purchased from American Type Culture Collection (Manassas, VA, USA), and cultured as monolayers at 37 °C in a humidified atmosphere of 5% CO_2_, and in Roswell Park Memorial Institute 1640 medium (RPMI-1640, Thermo Fisher Scientific, Waltham, MA, USA) supplemented with 10% heat-inactivated fetal bovine serum and 100 U/mL penicillin/streptomycin. Cells were exposed to escalating doses of gefitinib (Selleckchem, Houston, TX, USA) to induce resistance, and then routinely cultured in 1 μM gefitinib to maintain resistance.

### Lentivirus construction and infection

Short hairpin RNA (shRNA) against ANXA5 was obtained from RNAi Consortium (Broad Institute, Cambridge, MA, USA). Lentiviral plasmids (GV112) expressing shANXA5 or negative control shRNA were obtained from GeneChem (Shanghai, China). Lentiviral vector (GV358) overexpressing human EGFR (EGFR-over) (GenBank: NM_005228) was purchased from GeneChem. PC9R and H4006 cells grown to 50–70% confluence in 6-well plates were then infected at multiplicity of infection 10 with lentiviral particles produced in HEK 293 T cells and suspended in 4 μg/mL polybrene. After 24 h, cells were exposed to an escalating dose of gefitinib.

### Cell proliferation

Cells were seeded in 96-well plates at approximately 1000 cells/well in 200 μL culture medium. After 24 h, cells were stained for 1 h at 37 °C with 10 μL CCK8 reagent (Dojindo Laboratories, Kumamoto, Japan), and assayed at 450 nm on a Multiskan microplate reader (Thermo Fisher Scientific).

### EdU incorporation

PC9R and H4006R cells were incubated with 10 μM EdU (Thermo Fisher Scientific) for 4 h, fixed for 15 min at 20–25 °C with 3.7% formaldehyde in phosphate-buffered saline (PBS), processed according to the manufacturer’s instructions, counterstained with Hoechst 33,342, and imaged on an A1R confocal laser scanning microscope (Nikon, Tokyo, Japan). The total number of cells, as well as the number of EdU-stained cells, was determined in ImageJ v. 1.42 (National Institutes of Health, Bethesda, MD, USA).

### Detection of apoptotic cells by flow cytometry

Infected PC9R cells were seeded in 6-well plates at 5 × 10^5^ cells/well, treated with 1 μM gefitinib, digested with trypsin-EDTA (Thermo Fisher Scientific), washed three times with PBS, resuspended in 500 μL binding buffer, stained for 15 min at room temperature in the dark with 5 μL FITC-conjugated annexin V and 3 μL propidium iodide (Thermo Fisher Scientific), and sorted on a FACS Aria II flow cytometer (BD Biosciences, Franklin Lakes, NJ, USA).

### Cell cycle analysis

Infected PC9R cells were seeded in 6-well plates at 5 × 10^5^ cells/well, treated with 1 μM gefitinib, harvested, washed with PBS, fixed for 24 h at 4 °C in 70% ethanol, stained with propidium iodide for 30 min at room temperature in the dark, and analyzed by flow cytometry.

### Measurement of mitochondrial membrane potential

Mitochondrial membrane potential was measured using MitoProbe, which is based on tetraethylbenzimidazolylcarbocyanine iodide (JC-1) (Thermo Fisher Scientific), a compound that forms aggregates emitting red fluorescence at 590 nm in intact mitochondria, but is a monomer emitting green fluorescence at 490 nm in depolarized mitochondria. Thus, an increase in green fluorescence, as measured by flow cytometry, is indicative of mitochondrial damage.

### qPCR

Total RNA was extracted using TRIzol (Thermo Fisher Scientific), and reverse transcribed into cDNA using a reverse transcriptase (Toyobo, Osaka, Japan). Subsequently, 20 ng cDNA was used as qPCR template to quantify ANXA5 with 5’-ACCCTCTCGGCTTTATGATG-3′ and 5’-GATGGCTCTCAGTTCTTCAGG-3′, BIRC5 with 5’-AGAGTCCCTGGCTCCTCTA-3′ and 5’-CCCGTTTCCCCAATGACTTA-3′, PLK1 with 5’-GACAAGTACGGCCTTGGGTA-3′ and 5’-GTGCCGTCACGCTCTATGTA-3′, TOP2α with 5’-AGCAGATTAGCTTCGTCAACAGC-3′ and 5’-ACATGTCTGCCGCCCTTAGA-3′, CDK1 with 5’-TAGCGCGGATCTACCATACC-3′ and 5’-CATGGCTACCACTTGACCTG-3′, EGFR with 5′- TGTCCCCACGGTACTTACTCC-3′ and 5’-CCAAATGCTGATGAATCCAATG-3′, and β-actin with 5’-CTGGCACCCAGCACAATG-3′ and 5’-CCGATCCACACGGAGTACTTG-3′. Targets were amplified over 40 cycles at 95 °C for 15 s, 60 °C for 15 s, and 72 °C for 45 s. Gene expression was normalized to β-actin according to the cycle threshold (2^−ΔΔCT^) method.

### Western blotting

Total protein was extracted in radioimmunoprecipitation buffer (Beyotime Institute of Biotechnology, Shanghai, China), separated on polyacrylamide gels, transferred to polyvinylidene difluoride, and probed overnight at 4 °C with antibodies against ANXA5 (Abcam, Cambridge, UK), EGFR, PLK1, cyclin-dependent kinase (CDK)1, topoisomerase 2α (TOP2α), and baculoviral inhibitor of apoptosis repeat-containing 5 (BIRC5, also called survivin), cleaved caspase-3 (Asp175), cleaved PARP (Asp214), and actin (Cell Signaling Technology, Danvers, MA, USA). Membranes were then labeled at room temperature for 1 h with goat anti-rabbit or anti-mouse immunoglobulin G conjugated to horseradish peroxidase, visualized by enhanced chemiluminescence (Pierce, Rockford, IL, USA), and analyzed in Quantity One v.4.6 (Bio-Rad, Hercules, CA, USA).

### cDNA array screening

Total RNA was extracted using RNeasy Plus Mini Kit (Qiagen, Valencia, CA, USA) from PC9R cells expressing shANXA5 or negative control shRNA, reverse transcribed, and amplified using OneArray Plus RNA Amplification Kit (Phalanx Biotech Group, Taiwan). Cy5-labeled amplicons were hybridized to Human Whole Genome OneArray (Phalanx Biotech Group), imaged on a G2505C Agilent Microarray Scanner (Agilent Technologies, Santa Clara, CA, USA), and analyzed in Resolver (Rosetta Biosoftware, Seattle, WA, USA).

### Tumorigenicity

Animal experiments were approved by the Institutional Animal Care and Use Committee at Zhongshan Hospital of Fudan University, China. Male BALB/c nude mice 4–6 weeks old were obtained from Shanghai Experimental Animal Center, Chinese Academy of Sciences (Shanghai, China), and were subcutaneously injected in the right flank with PC9R cells. Tumor volume was calculated as volume = (length × width^2^)/2. After 1 month, tumors were dissected, weighed, sectioned in paraffin, and analyzed by immunohistochemistry for Ki67 to detect proliferating cells. Ki67-stained cells in randomly selected fields from each tissue section were quantified in ImageJ.

### Pleural effusion sample collection

A total of 27 lung adenocarcinoma patients, including 18 who were sensitive to and nine who had acquired resistance to EGFR TKIs, were enrolled in the study at the Department of Pulmonary Medicine, Zhongshan Hospital, Fudan University. All study participants provided informed consent and the study protocol was approved by the institutional ethics committee (approval number B2016-154R). All patient managements and pleural effusion sample collection were carried out in accordance with the relevant guidelines. Pleural effusions were collected and centrifuged at 1000×*g* for 10 min. Cell pellets were washed twice with PBS and total RNA was extracted from cells using TRIzol reagent (Invitrogen). *ANXA5* mRNA level was quantitated by qPCR.

### Statistical analysis

Data are reported as mean ± standard deviation (SD) of at least three independent experiments with three replicates. Differences among multiple groups were evaluated by one-way analysis of variance followed by Bonferroni’s multiple comparisons test, while Student’s t test was used to compare two groups. Differences were considered statistically significant at *P* < 0.05.

## Results

### ANXA5 is upregulated in gefitinib-resistant cells and confers gefitinib resistance

**Fig. 1:**
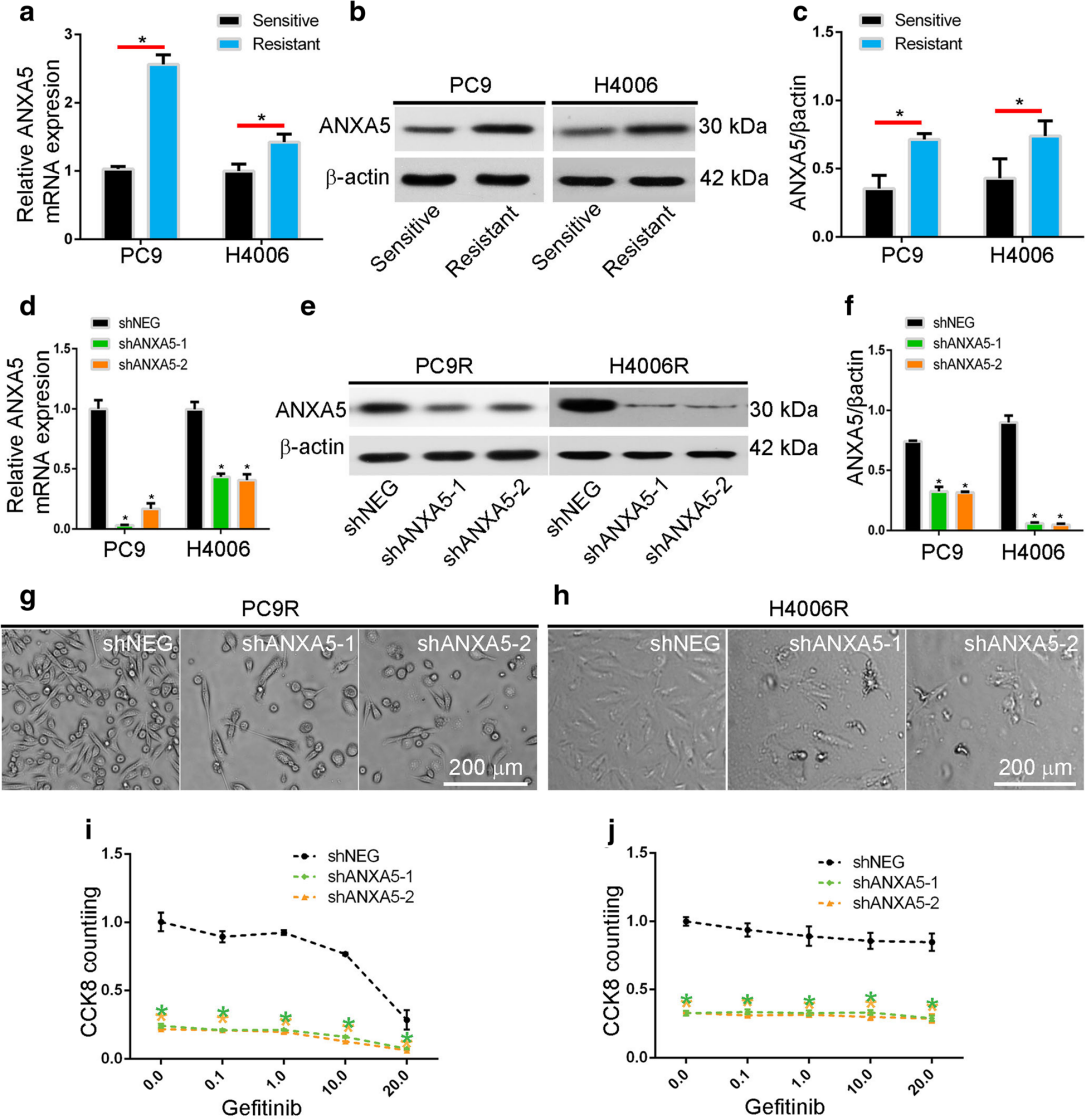
ANXA5 is upregulated in gefitinib-resistant cells, and confers gefitinib resistance. **a**-**c** ANXA5 expression in PC9R and H4006R cells, as assessed by qPCR and western blot. **d**-**f** qPCR and western blot of ANXA5 expression in PC9R and H4006R cells expressing shANXA5 or negative control shRNA (shNEG). **g**-**h** Morphology of PC9R and H4006R cells expressing shANXA5 or shNEG and cultured in 1 μM gefitinib. **i**-**j** Viability of PC9R and H4006R cells expressing shANXA5 or shNEG and treated with different concentrations of gefitinib, as determined by CCK8 assay. Data are representative of at least three similar experiments. *, *P* < 0.05 vs. negative control

To investigate the function of ANXA5, we generated gefitinib-resistant PC9R and H4006R lung cancer cells by exposure to escalating doses of gefitinib. We found, by quantitative PCR and western blot, that ANXA5 is upregulated in gefitinib-resistant cells in comparison to parental, gefitinib-sensitive cells, implying that the gene may promote gefitinib resistance (Fig. 1a-c).

To test this possibility, gefitinib-resistant PC9R and H4006R cells were infected for 24 h with viral particles expressing shRNA against ANXA5. qPCR and western blots showed that ANXA5 was efficiently knocked down in both cell lines (Fig. 1d-f), relative to cells expressing negative control shRNA. Knockdown of ANXA5 also inhibited cell proliferation in 1 μM gefitinib, as assessed by cell density after 96 h (Fig. 1g & h). Finally, we found that knockdown of ANXA5 reduced cell viability in 0.1–20.0 μM gefitinib, as assessed by Cell Counting Kit 8 (CCK8) assay (Fig. 1i & j).

### ANXA5 silencing promotes G2/M arrest and apoptosis in gefitinib-resistant cells through caspase activation and mitochondrial damage

**Fig. 2:**
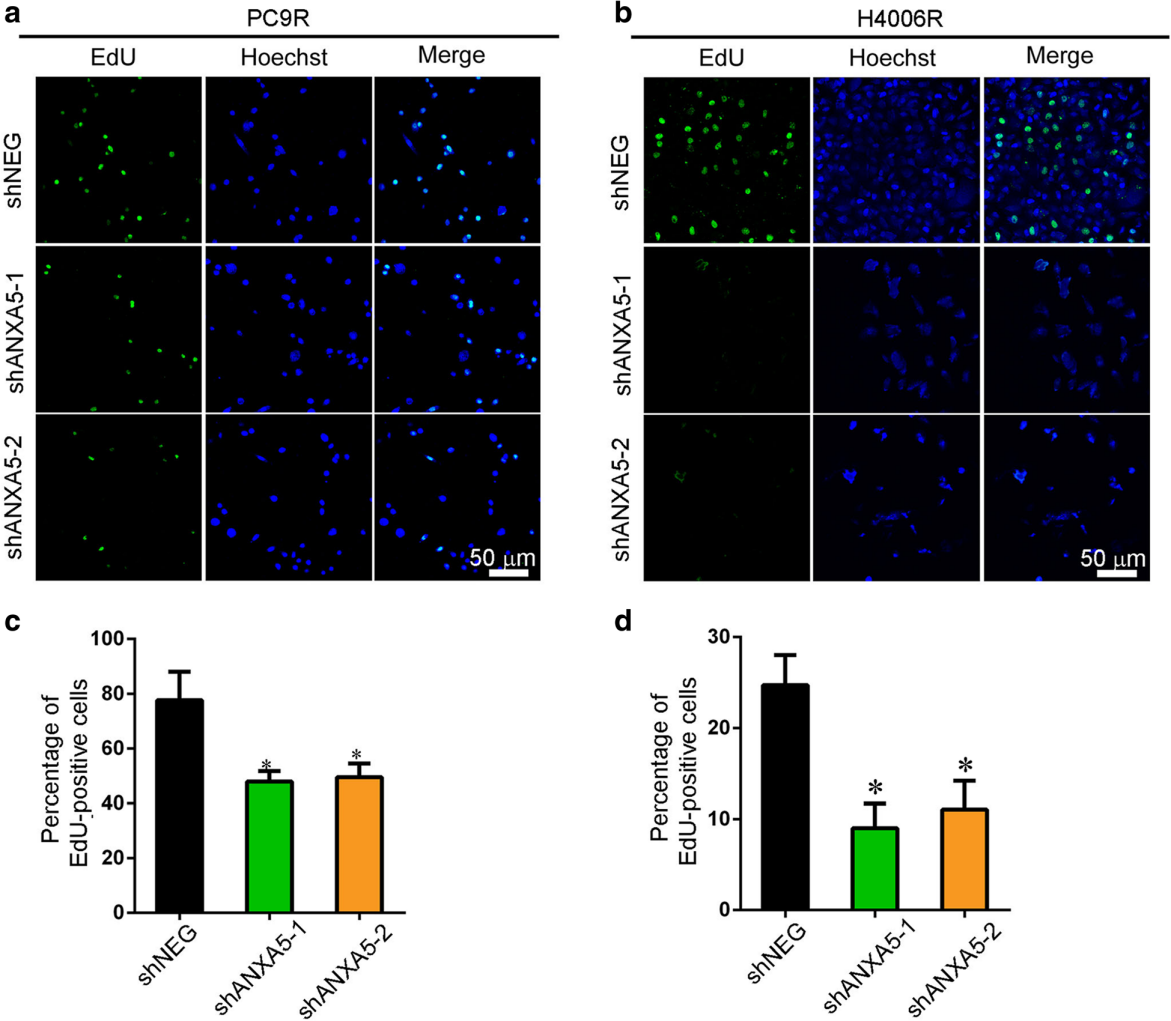
Knockdown ANXA5 inhibits proliferation of gefitinib-resistant cells. **a**-**b** PC9R and H4006R cells expressing ANXA5 shRNA or shNEG were treated with 1 μM gefitinib and then stained with EdU and Hoechst 33,342. **c**-**d** EdU-stained cells were more abundant in control cells than in ANXA5-depleted cells. Data are representative of at least three similar experiments. *, *P* < 0.05 vs. negative control

**Fig. 3:**
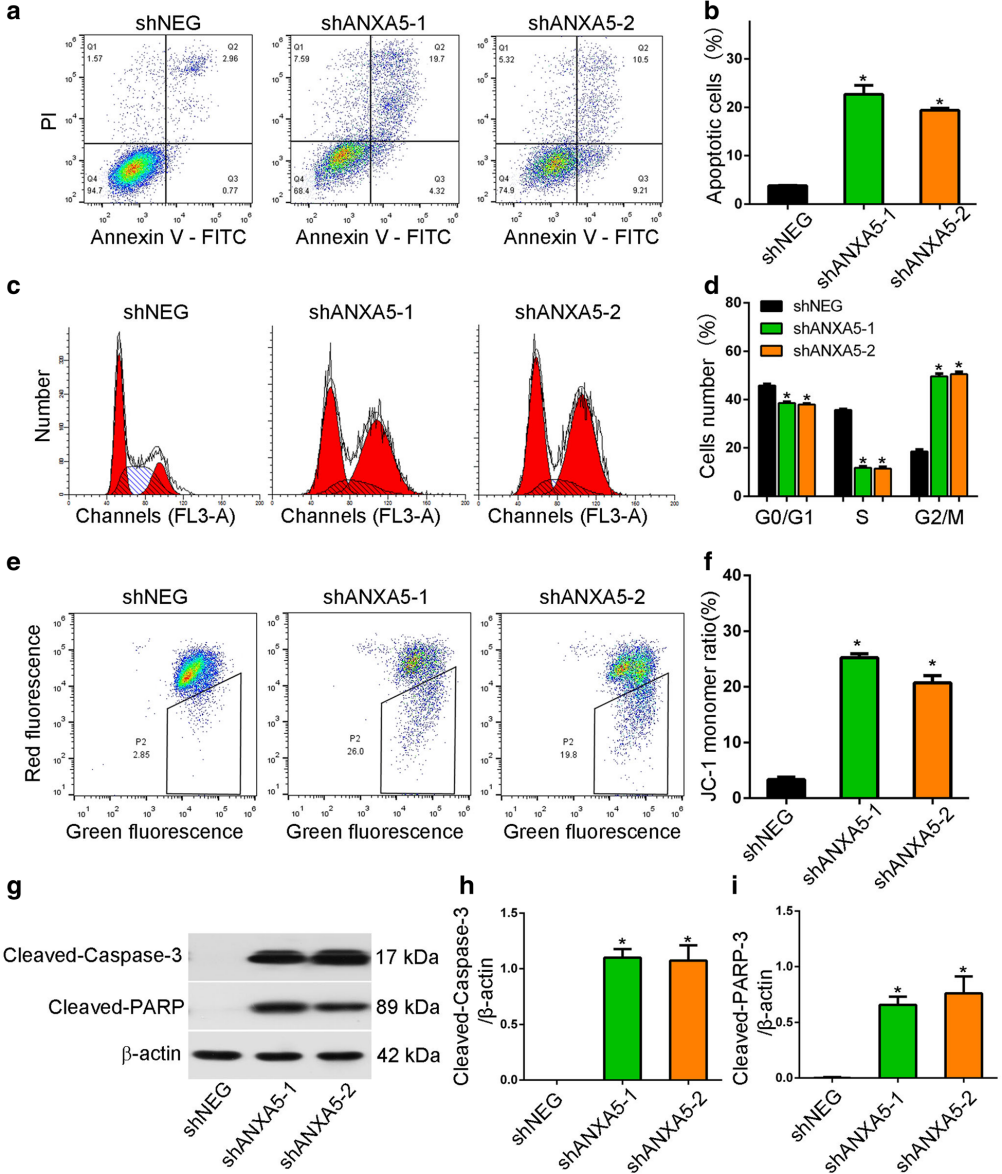
ANXA5 silencing promotes G2/M arrest and apoptosis in gefitinib-resistant cells through caspase activation and mitochondrial damage. **a**-**b** PC9R cells expressing ANXA5 shRNA or shNEG were treated with 1 μM gefitinib, stained with FITC-annexin V/propidium iodide, and then analyzed by flow cytometry. ANXA5 silencing increased apoptosis. **c**-**d** PC9R cells expressing ANXA5 shRNA or shNEG were treated with 1 μM gefitinib, and cell cycle distribution was assessed by flow cytometry. **e**-**f** Representative histograms of JC-1 staining as measured by flow cytometry. ANXA5 silencing increased the proportion of monomeric JC-1 in ANXA5-depleted cells, following incubation with 1 μM gefitinib. **g**-**i** Western blot of cleaved caspase-3 and cleaved PARP in PC9R cells expressing shANXA5 or shNEG. Data are representative of three similar experiments. *, *P* < 0.05 vs. negative control

We confirmed, by 5-ethynyl-2′-deoxyuridine (EdU) staining (Fig. 2a & b), that ANXA5 knockdown suppresses the proliferation of gefitinib-resistant cells in the presence of gefitinib (Fig. 2c & d), implying that ANXA5 promotes proliferation in gefitinib-resistant cells. Apoptosis was also enhanced following ANXA5 knockdown and exposure to 1 μM gefitinib, as assessed by annexin V-fluorescein isothiocyanate (FITC)/propidium iodide staining and flow cytometry (Fig. 3a & b). Since apoptosis is intimately associated with the cell cycle, we also evaluated the effect of ANXA5 on the cell cycle distribution of gefitinib-resistant cells. We found that the number of cells in the G0/G1 and S phase decreased upon ANXA5 silencing, while cells in the G2/M phase accumulated, indicating cell cycle arrest (Fig. 3c & d). We also assessed mitochondrial integrity by staining cells with JC-1, a dye that emits red fluorescence as aggregates in intact mitochondria, but emits green fluorescence as monomers in damaged mitochondria. We found that, in the presence of 1 μM gefitinib, the proportion of monomeric JC-1 was higher in ANXA5-silenced PC9R cells than in control cells (Fig. 3e & f). Moreover, the cleaved, apoptotic forms of caspase-3 and poly-ADP-ribose polymerase accumulated in ANXA5-depleted cells relative to control cells (Fig. 3g-i).

### ANXA5 knockdown suppresses the proliferation of gefitinib-resistant cells in vivo

**Fig. 4:**
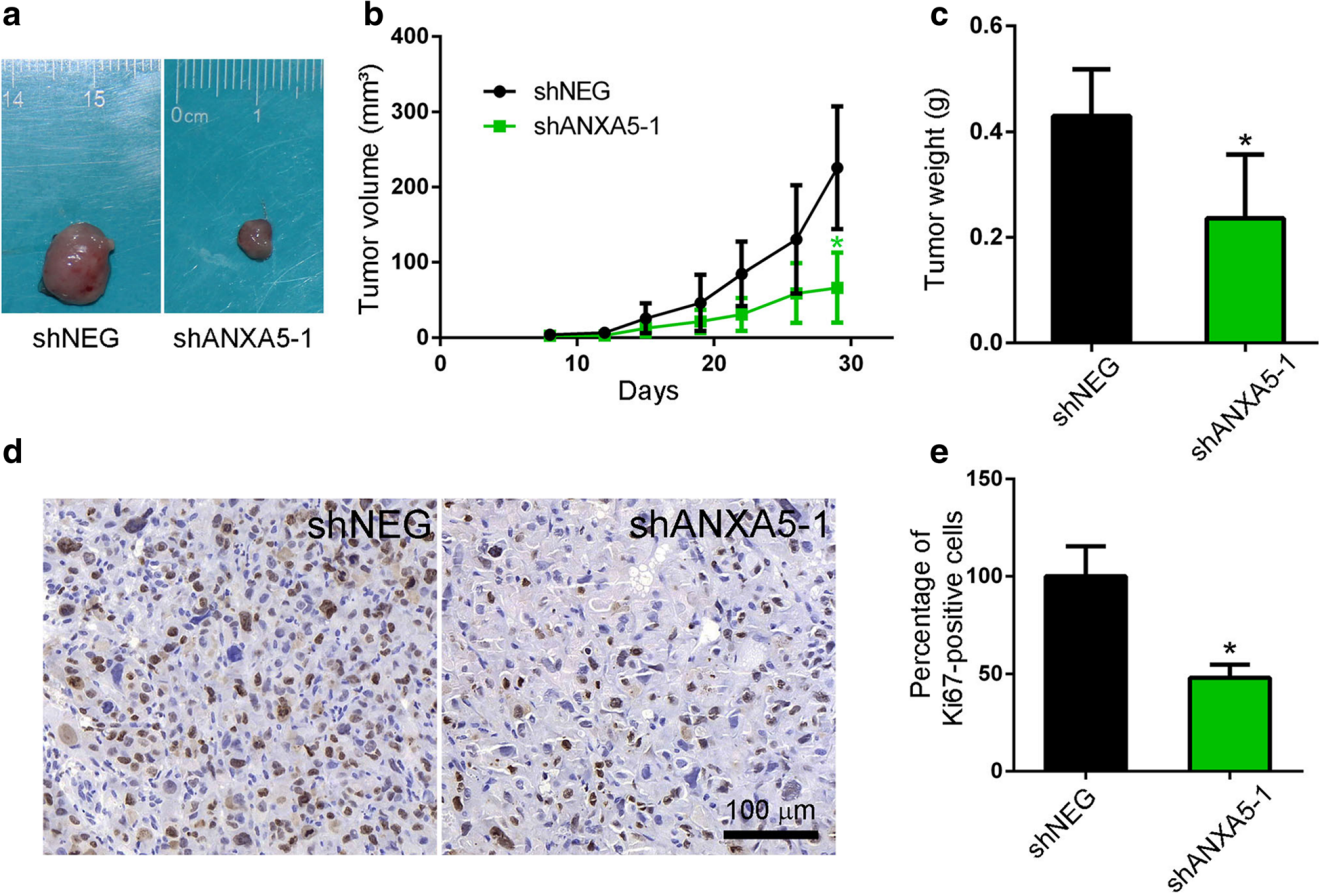
ANXA5 knockdown suppresses tumor cell proliferation in vivo. **a** Representative images of tumors dissected from animals one month after injection of PC9R cells expressing shANXA5 or negative control (shNEG). **b** Tumor volume was measured twice a week. **c** Tumor weight. **d** Representative Ki67 expression in xenograft tumors. **e** Ki67-positive cells were quantified in randomly selected fields, *n* = 5 mice per group. *, *P* < 0.05 vs. negative control

ANXA5-silenced and control gefitinib-resistant PC9R cells were injected into nude mice to assess tumorigenicity in vivo. Visible tumors were observed in animals after 1 week, although the volume of tumors derived from ANXA5-silenced cells was significantly smaller than that of tumors from control cells (Fig. 4a). A similar trend was observed after 1 month (Fig. 4b & c). Accordingly, the abundance of proliferating, Ki67-positive cells were lower in ANXA5-deficient tumors than in control xenografts (Fig. 4d & e).

### ANXA5 knockdown induces G2/M arrest via PLK1

**Fig. 5:**
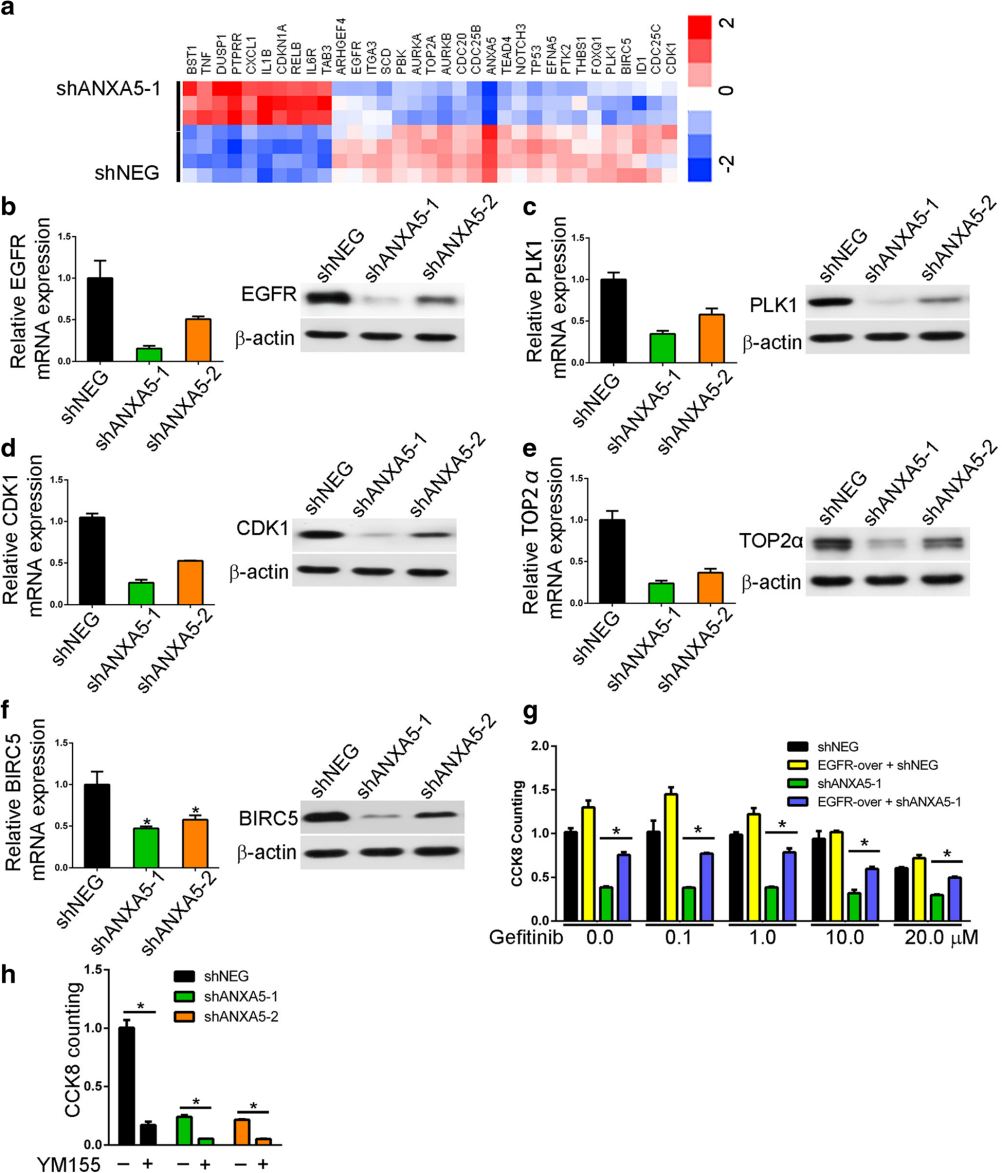
Knockdown ANXA5 induces G2/M arrest by suppressing polo-like kinase 1. **a** Whole-genome human cDNA array screening. **b** mRNA and protein levels of EGFR, polo-like kinase 1 (**c**), cyclin-dependent kinase 1 (**d**), topoisomerase 2α (**e**), and BIRC5 (**f**) were determined by qPCR and western blot in PC9R cells expressing shANXA5 or negative control shRNA (shNEG). **g** Proliferation of PC9R cells co-transfected with EGFR (EGFR-over), shANXA5, and shNEG, as determined by CCK8. **h** Proliferation of PC9R cells transfected with shANXA5 and shNEG and treated with the BIRC5 inhibitor YM155, as determined by CCK8. *, *P* < 0.05 vs. negative control

Using whole-genome human cDNA array, we found that ANXA5 depletion altered the expression of cell cycle molecules. qPCR analysis also confirmed that EGFR, polo-like kinase (PLK) 1, cyclin-dependent kinase (CDK)1, topoisomerase 2α (TOP2α), and baculoviral inhibitor of apoptosis repeat-containing 5 (BIRC5, also called survivin) were significantly downregulated (Fig. 5a-f). These results imply that ANXA5 knockdown elicits G2/M arrest in gefitinib-resistant cells by suppressing polo-like kinase 1, which triggers progression from G2 to mitosis in normal conditions via a phosphorylation cascade from cell division control protein 25 to Wee1 kinase, CDK1, TOP2α, and BIRC5 [15].

Furthermore, we found that overexpression of EGFR partly reversed the apoptotic effects of ANXA5 depletion in PC9R cells exposed to 1 μM gefitinib (Fig. 5g), confirming that ANXA5 induces drug resistance by interfering with EGFR signaling. YM155, a BIRC5 inhibitor in clinical trial as an anticancer agent, also decreased the proliferation of PC9R cells relative to the negative control, especially in cells transfected with shANXA5 (Fig. 5h).

### ANXA5 overexpression is associated with clinical resistance to EGFR tyrosine kinase inhibitors

**Fig. 6:**
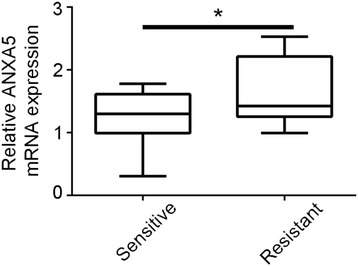
ANXA5 overexpression increases resistance to EGFR tyrosine kinase inhibitors in lung cancer patients. **a** Scatterplot of *ANXA5* mRNA expression in lung adenocarcinomas sensitive or resistant to EGFR tyrosine kinase inhibitors. *, *P* < 0.05 vs. negative control

Cell pellet, which includes cancer cells, was collected from pleural effusions in 27 patients with lung adenocarcinomas, of whom 18 were sensitive and nine were resistant to EGFR tyrosine kinase inhibitors. ANXA5 was downregulated in the former relative to the latter (Fig. 6a), even though clinical characteristics, listed in Table 1, were comparable.

**Table 1:** The clinical characteristics of EGFR TKI-sensitive and –-resistance lung adenocarcinoma patients

	Clinicopathological Features	Number of cases	EGFR TKI-sensitive	EGFR TKI-resistance	*p* Value
Age (years)					
< 65		14	10	4	0.445
≥ 65		13	8	5	
Gender					
Male		10	5	5	0.162
Female		17	13	4	
Smoking					
No-smokers		20	14	6	0.622
Smokers		6	4	2	
Tumor size					
T1/T2		15	10	5	0.675
T3/T4		9	6	3	
lymphatic metastasis					
Absent		16	10	6	0.449
Present		11	8	3	
Clinical stage					
IV期		27	18	9	

*P* value represents the probability from a Chi-square test for different number of EGFR TKI-sensitive and –-resistance cases

## Discussion

First-generation EGFR tyrosine kinase inhibitors, including gefitinib and erlotinib, are the first-line treatment against advanced non-small cell lung cancers with EGFR activating mutations, especially in Asians, females, never smokers, and/or patients with adenocarcinoma [16]. However, resistance to such inhibitors is a serious issue, with approximately 20–30% of patients unresponsive to treatment. Even among patients who show initial improvement, progressive disease eventually develops about 1 year after treatment [17]. Therefore, understanding the mechanisms of resistance is essential to improve efficacy. A few such mechanisms have been identified, including a secondary T790 M mutation in exon 20 of EGFR, amplification of the *MET* proto-oncogene, and overexpression of hepatocyte growth factor [18–20]. Nevertheless, approximately 25% of resistant cases are not due to these mechanisms. We now report that ANXA5 is significantly upregulated in gefitinib-resistant cells, and that it promotes gefitinib resistance by inhibiting apoptosis and G2/M arrest via polo-like kinase 1.

EGFR activation promotes cancer cell division, survival, metastasis, and cellular repair. The major downstream signaling route includes Ras/Raf/mitogen-activated protein kinase, Janus kinase/signal transducer and activator of transcription, and phosphoinositide 3-kinase/AKT/mammalian target of rapamycin. EGFR tyrosine kinase inhibitors efficiently block these cascades and induce cell cycle arrest and cell apoptosis [21, 22]. Thus, escape from cell cycle arrest and apoptosis is an important feature of resistance to EGFR tyrosine kinase inhibitors.

ANXA5 is an important cell membrane protein that reseals damaged membranes by forming two-dimensional arrays at high Ca^2+^ concentrations [9]. As EGFR tyrosine kinase inhibitors may damage membranes via release of apoptotic proteins and induction of immunity [23], ANXA5 may confer resistance through membrane repair. Accordingly, gefitinib causes mitochondrial degradation in cells that were already resistant to EGFR tyrosine kinase inhibitors but were then depleted of ANXA5. ANXA5 knockdown also significantly enhanced apoptosis, consistent with the model that failure of membrane repair eventually causes apoptosis [24]. Moreover, we found that ANXA5 knockdown represses G2/M proteins, and thereby induces cell cycle arrest. For example, PLK 1, which promotes transition from G2 to mitosis by phosphorylating cell division control protein 25 and Wee1 kinase, was downregulated along with cyclin-dependent kinase 1, which is activated further downstream [25]. Loss of cyclin-dependent kinase 1 also downregulated its substrates BRIC5 and TOP 2α [26, 27], of which the former regulates microtubule dynamics at G2/M. Ultimately, loss of BRIC5 induces G2 arrest, activates caspase-3, and elicits apoptosis, as we observed [28, 29]. On the other hand, TOP 2α is abundantly expressed at G2/M to promote chromosome replication, and its loss potently triggers G2/M arrest [30, 31]. Collectively, our data show that ANXA5 knockdown induces G2/M arrest and apoptosis by suppressing polo-like kinase 1 signal pathwayin cells resistant to EGFR tyrosine kinase inhibitors.

## Conclusions

We report for the first time that ANXA5 is upregulated in gefitinib-resistant cells and tissues. Accordingly, knockdown of ANXA5 reduces gefitinib resistance by promoting apoptosis and G2/M arrest. Thus, ANXA5 is an important mediator of resistance to EGFR tyrosine kinase inhibitors, and is a potential therapeutic target in recalcitrant lung cancers.

## Abbreviations

ANXA5: Annexin A5
BIRC5: Baculoviral inhibitor of apoptosis repeat-containing 5
CCK: Cell Counting Kit
CDK1: Cyclin-dependent kinase 1
EdU: 5-ethynyl-2'′’-deoxyuridine
EGFR: Epidermal growth factor receptor
FITC: Fluorescein isothiocyanate
IHC: Immunohistochemistry
INM: Inner nuclear membrane
JC-1: Tetraethylbenzimidazolylcarbocyanine iodide
MAPK: Mitogen-activated protein kinase
NSCLC: Non-small cell lung cancer
PARP: Poly-ADP-ribose polymerase
PI: Propidium iodide
PI3K: Phosphoinositide 3-kinase
PLK1: Polo-like kinase 1
qPCR: Quantitative PCR
RPMI-1640: Roswell Park Memorial Institute 1640
SD: Standard deviation
sh: short hairpin
shNEG: Short hairpin negative control
STAT: Signal transducer and activator of transcription
TKIs: Tyrosine kinase inhibitors
TOP2α: Topoisomerase 2α
WB: Western blotting
